# Supplementary material for: End Sequence Analysis Toolkit (ESAT) expands the extractable information from single-cell RNA-seq data
Source: Genome Res. 2016 Oct;26(10):1397–410. doi: 10.1101/gr.207902.116 (PMC5052061; doi:10.1101/gr.207902.116)
Supplement: Supplemental Material [file supp_gr.207902.116_Supp_Materials.tar.gz › ESAT-esat_v0.1/doc/html/classbroad_1_1pda_1_1seq_1_1segmentation_1_1_generic_alignment_data_model.html]

ESAT: broad.pda.seq.segmentation.GenericAlignmentDataModel Class Reference


ESAT


- Main Page
- Related Pages
- Packages
- Classes
- Files

- Class List
- Class Index
- Class Hierarchy
- Class Members

All Classes Namespaces Files Functions Variables Enumerator Pages

- broad
- pda
- seq
- segmentation
- GenericAlignmentDataModel

Classes |
Public Member Functions |
List of all members

broad.pda.seq.segmentation.GenericAlignmentDataModel Class Reference

Inheritance diagram for broad.pda.seq.segmentation.GenericAlignmentDataModel:

[legend]

Collaboration diagram for broad.pda.seq.segmentation.GenericAlignmentDataModel:

[legend]

|  |  |
| --- | --- |
| Classes | |
| class | **EmptyCloseableIterator** |
|  | |

|  |  |
| --- | --- |
| Public Member Functions | |
|  | GenericAlignmentDataModel (String filename, String lengthFile, boolean upweightSplices, double minMappingQuality, boolean removeDuplicatesFlag, boolean weighReadCounts, String strand, boolean loadPairsAsFragments) throws IOException |
|  | |
|  | GenericAlignmentDataModel (String filename, String lengthFile, boolean upweightSplices, double minMappingQuality) throws IOException |
|  | |
|  | GenericAlignmentDataModel (String filename, String lengthFile) throws IOException |
|  | |
|  | GenericAlignmentDataModel (String filename, String lengthFile, int minMappingQuality) throws IOException |
|  | |
|  | GenericAlignmentDataModel (String filename, String lengthFile, boolean upweightSplices) throws IOException |
|  | |
|  | GenericAlignmentDataModel (String filename, String lengthFile, boolean upweightSplices, int minMappingQuality, boolean removeDuplicatesFlag) throws IOException |
|  | |
|  | GenericAlignmentDataModel (String filename, String lengthFile, int minMappingQuality, boolean removeDuplicatesFlag) throws IOException |
|  | |
|  | GenericAlignmentDataModel (String filename, String lengthFile, boolean removeDuplicatesFlag, boolean weighReadCounts) throws IOException |
|  | |
|  | GenericAlignmentDataModel (String filename, String lengthFile, boolean upweightSplices, boolean removeDuplicatesFlag, boolean weighReadCounts) throws IOException |
|  | |
|  | GenericAlignmentDataModel (String filename, String lengthFile, boolean upweightSplices, int minMappingQuality, boolean removeDuplicatesFlag, boolean weighReadCounts) throws IOException |
|  | |
|  | GenericAlignmentDataModel (String filename, String lengthFile, boolean upweightSplices, int minMappingQuality, boolean removeDuplicatesFlag, boolean weighReadCounts, String strand) throws IOException |
|  | |
| CloseableIterator< Alignment > | getAnnotationOverlappingRegion (Annotation align) throws IOException |
|  | |
| double | getBasesCoveredPerAlignment (Gene gene, IntervalTree< Alignment > tree, int EF) |
|  | |
| double | getCountsPerAlignmentFullyContained (Gene gene, IntervalTree< Alignment > tree, int EF) |
|  | |
| int | getBasesCovered (Annotation region, int EF) throws IOException |
|  | |
| Gene | getPeak (Gene gene, Gene startCodon, IntervalTree< Alignment > tree, int EF) |
|  | |
| Map< String, Integer > | getChrLengths () |
|  | |
| int | getChrLength (String chr) |
|  | |
| CloseableIterator< Alignment > | getChrReadIterator (String chromosome) throws IOException |
|  | |
| int | getChunkEnd () |
|  | |
| int | getChunkStart () |
|  | |
| double | getCounts (String chr) throws IOException |
|  | |
| double | getCountsOfUniqueOverlappers (Annotation target, Annotation exclude, IntervalTree< Alignment > tree, int EF) |
|  | |
| double | getCountsOfUniqueOverlappers (Annotation target, Gene exclude, IntervalTree< Alignment > tree, int EF) |
|  | |
| double | getCountsOnBase (String chr, int index) throws IOException |
|  | |
| double | getCountsOnWindow (String chr, int startIndex, int endIndex) throws IOException |
|  | |
| double | getCountsPerAlignmentUncached (String chr, int startIndex, int endIndex) throws IOException |
|  | |
| double | getCountsPerAlignment (Annotation align, int EF) throws IOException |
|  | |
| double | getCountsPerAlignment (Annotation align) throws IOException |
|  | |
| double | getCountsPerAlignment (Annotation align, IntervalTree< Alignment > tree, int EF) |
|  | |
| boolean | loadedPairsAsFragments () |
|  | |
| double | getCountsPerAlignmentForPolyA (Annotation align, int EF) throws IOException |
|  | |
| double | getCountsPerAlignment (Gene gene, IntervalTree< Alignment > tree, int EF) |
|  | |
| double | getCountsPerAnnotationtranded (Gene gene, IntervalTree< Alignment > tree, int EF, String orientation) |
|  | |
| double | getCountsPerAlignment (Annotation[] Annotation, IntervalTree< Alignment > tree, int EF) |
|  | |
| double | getCountsPerAlignment (Annotation[] Annotation, int EF) throws IOException |
|  | |
| double | getCountsPerAlignment (Annotation align, Map< String, IntervalTree< Annotation >> goodExonTree, IntervalTree< Alignment > tree, int extensionFactor) |
|  | |
| double | getCountsPerAlignmentWithSameEndpointForPolyA (Annotation align, int EF, boolean polyAIsOnRight, int typicalOriginalAlignmentLength, int maxDistanceFromOriginalAlignmentEndpoint) throws IOException |
|  | |
| int | getCountsWithinExons (Annotation align, IntervalTree< Alignment > tree, int EF) |
|  | |
| int | getCountsWithinExons (Annotation align, Iterator< Alignment > iter, int EF) |
|  | |
| Iterator< Annotation > | getExonAnnotationOverlappingRegion (Annotation align) throws IOException |
|  | |
| Iterator< Annotation > | getExonAnnotationOverlappingRegion (IntervalTree< Alignment > tree) |
|  | |
| IntervalTree< Annotation > | getFullIntervalTreeAsAnnotation (String chr) throws IOException |
|  | |
| GeneCounts | getGeneCounts (Gene gene, int extensionFactor) throws IOException |
|  | |
| String | getModelFilePath () |
|  | |
| IntervalTree< Alignment > | getIntervalTree (String chr, int start, int end) throws IOException |
|  | |
| IntervalTree< Alignment > | getIntervalTreeCached (String chr, int start, int end) throws IOException |
|  | |
| IntervalTree< Alignment > | getIntervalTreeTruncated (String chr, int start, int end) throws IOException |
|  | |
| IntervalTree< Alignment > | getIntervalTreeTruncatedCached (String chromosome, int start, int end) throws IOException |
|  | |
| double | getMinimumMappingQuality () |
|  | |
| double | getNumberOfSplicedReads (Annotation align) throws IOException |
|  | |
| double | getNumberOfSplicedReads () |
|  | |
| Collection< Annotation > | getOverlappingRegions (String chr) throws IOException |
|  | |
| CloseableIterator< Alignment > | getReadIterator () |
|  | |
| CloseableIterator< Alignment > | getReadIterator (Annotation region) throws IOException |
|  | |
| SAMFileHeader | getSamHeader () throws IOException |
|  | |
| Collection< Annotation > | getSplicedReadExons (String chr) throws IOException |
|  | |
| void | setExtensionFactor (int extensionFactor) |
|  | |
| Map< Annotation, Integer > | getSplicedReads (Annotation align) throws IOException |
|  | |
| Map< Annotation, Integer > | getSplicedReads (Annotation align, final int minIntronSize, final int maxIntronSize, int minNumIntrons, Sequence chrSeq) throws IOException |
|  | |
| Map< Annotation, Integer > | getSplicedReads (Annotation align, final int minIntronSize, final int maxIntronSize) throws IOException |
|  | |
| Map< Annotation, Integer > | getSplicedReads (Annotation region, Predicate< Annotation > filter) throws IOException |
|  | |
| Map< Annotation, Integer > | getSplicedReads (Annotation region, Collection< Predicate< Annotation >> filters, int minNumIntrons) throws IOException |
|  | |
| Map< Annotation, Integer > | getSplicedReads (Annotation region, Collection< Predicate< Annotation >> filters, int minNumIntrons, Sequence chrSeq) throws IOException |
|  | |
| Map< Annotation, Integer > | getSplicedReads (Annotation region, Collection< Predicate< Annotation >> filters) throws IOException |
|  | |
| Map< Annotation, Integer > | getSplicedReads (Annotation region, Collection< Predicate< Annotation >> filters, Sequence chrSeq) throws IOException |
|  | |
| Map< Annotation, Integer > | getSplicedReads (int minIntronSize, int maxIntronSize) throws IOException |
|  | |
| double | getSpliceWeightFactor () |
|  | |
| double[] | getTotalNumberOfMappedReads () |
|  | |
| double[] | getTotalNumberOfMappedStrandedReads () |
|  | |
| double | getTotalNumberOfStrandedReads () |
|  | |
| double | getTotalNumberOfReads () |
|  | |
| double | getWeight () throws IOException |
|  | |
| void | setChunkSize (int chunkSize) |
|  | |
| void | setMinimumMappingQuality (double minimumMappingQual) |
|  | |
| void | setNegativeStranded () |
|  | |
| void | setSecondRead () |
|  | |
| void | setFirstRead () |
|  | |
| void | setNormalizationStrategy (ReadCountNormalizer normalizer) |
|  | |
| void | setPositiveStranded () |
|  | |
| boolean | hasNextExon (String chr) throws IOException |
|  | |
| boolean | isPositiveStranded () |
|  | |
| boolean | isNegativeStranded () |
|  | |
| boolean | isStranded () |
|  | |
| boolean | passes (Gene gene, IntervalTree< Alignment > tree, int EF, double cutoff) |
|  | |
| long | passesCutoff (Annotation align, IntervalTree< Alignment > tree, int EF, double threshold) |
|  | |
| void | resetTreeCache (String chr) |
|  | |
| void | resetTreeCache () |
|  | |
| void | restartIterator () |
|  | |
| void | unsetStranded () |
|  | |
| Gene | updateGeneByFirstCounts (Gene gene, IntervalTree< Alignment > tree, int EF) |
|  | |
| void | setRemoveDuplicatesFlag (boolean flag) |
|  | |
| void | setWeighReadCountsFlag (boolean flag) |
|  | |
| double | countReads (Alignment record) |
|  | |
| Map< String, Integer > | getChromosomeLengths () |
|  | |
| int | getChromosomeLength (String chr) |
|  | |
| CloseableIterator< Alignment > | getChromosomeReadIterator (String chromosome) throws IOException |
|  | |
| double | getScorePerAlignmentFromCache (Gene window, IntervalTree< Alignment > chunkAlignmentTree, int extensionFactor) |
|  | |
| void | clearFullIntervalTreeAsAlignmentsCached (String chr) |
|  | |
| Public Member Functions inherited from broad.pda.seq.segmentation.AlignmentDataModel | |
| Map< Annotation, Integer > | getSplicedReads (Annotation align, int minIntronSize, int maxIntronSize) throws IOException |
|  | |

## Constructor & Destructor Documentation

|  |  |  |  |
| --- | --- | --- | --- |
| broad.pda.seq.segmentation.GenericAlignmentDataModel.GenericAlignmentDataModel | ( | String | *filename*, |
|  |  | String | *lengthFile*, |
|  |  | boolean | *upweightSplices*, |
|  |  | double | *minMappingQuality*, |
|  |  | boolean | *removeDuplicatesFlag*, |
|  |  | boolean | *weighReadCounts*, |
|  |  | String | *strand*, |
|  |  | boolean | *loadPairsAsFragments* |
|  | ) |  | throws IOException |

Here is the call graph for this function:

|  |  |  |  |
| --- | --- | --- | --- |
| broad.pda.seq.segmentation.GenericAlignmentDataModel.GenericAlignmentDataModel | ( | String | *filename*, |
|  |  | String | *lengthFile*, |
|  |  | boolean | *upweightSplices*, |
|  |  | double | *minMappingQuality* |
|  | ) |  | throws IOException |

|  |  |  |  |
| --- | --- | --- | --- |
| broad.pda.seq.segmentation.GenericAlignmentDataModel.GenericAlignmentDataModel | ( | String | *filename*, |
|  |  | String | *lengthFile* |
|  | ) |  | throws IOException |

|  |  |  |  |
| --- | --- | --- | --- |
| broad.pda.seq.segmentation.GenericAlignmentDataModel.GenericAlignmentDataModel | ( | String | *filename*, |
|  |  | String | *lengthFile*, |
|  |  | int | *minMappingQuality* |
|  | ) |  | throws IOException |

|  |  |  |  |
| --- | --- | --- | --- |
| broad.pda.seq.segmentation.GenericAlignmentDataModel.GenericAlignmentDataModel | ( | String | *filename*, |
|  |  | String | *lengthFile*, |
|  |  | boolean | *upweightSplices* |
|  | ) |  | throws IOException |

|  |  |  |  |
| --- | --- | --- | --- |
| broad.pda.seq.segmentation.GenericAlignmentDataModel.GenericAlignmentDataModel | ( | String | *filename*, |
|  |  | String | *lengthFile*, |
|  |  | boolean | *upweightSplices*, |
|  |  | int | *minMappingQuality*, |
|  |  | boolean | *removeDuplicatesFlag* |
|  | ) |  | throws IOException |

|  |  |  |  |
| --- | --- | --- | --- |
| broad.pda.seq.segmentation.GenericAlignmentDataModel.GenericAlignmentDataModel | ( | String | *filename*, |
|  |  | String | *lengthFile*, |
|  |  | int | *minMappingQuality*, |
|  |  | boolean | *removeDuplicatesFlag* |
|  | ) |  | throws IOException |

|  |  |  |  |
| --- | --- | --- | --- |
| broad.pda.seq.segmentation.GenericAlignmentDataModel.GenericAlignmentDataModel | ( | String | *filename*, |
|  |  | String | *lengthFile*, |
|  |  | boolean | *removeDuplicatesFlag*, |
|  |  | boolean | *weighReadCounts* |
|  | ) |  | throws IOException |

|  |  |  |  |
| --- | --- | --- | --- |
| broad.pda.seq.segmentation.GenericAlignmentDataModel.GenericAlignmentDataModel | ( | String | *filename*, |
|  |  | String | *lengthFile*, |
|  |  | boolean | *upweightSplices*, |
|  |  | boolean | *removeDuplicatesFlag*, |
|  |  | boolean | *weighReadCounts* |
|  | ) |  | throws IOException |

|  |  |  |  |
| --- | --- | --- | --- |
| broad.pda.seq.segmentation.GenericAlignmentDataModel.GenericAlignmentDataModel | ( | String | *filename*, |
|  |  | String | *lengthFile*, |
|  |  | boolean | *upweightSplices*, |
|  |  | int | *minMappingQuality*, |
|  |  | boolean | *removeDuplicatesFlag*, |
|  |  | boolean | *weighReadCounts* |
|  | ) |  | throws IOException |

|  |  |  |  |
| --- | --- | --- | --- |
| broad.pda.seq.segmentation.GenericAlignmentDataModel.GenericAlignmentDataModel | ( | String | *filename*, |
|  |  | String | *lengthFile*, |
|  |  | boolean | *upweightSplices*, |
|  |  | int | *minMappingQuality*, |
|  |  | boolean | *removeDuplicatesFlag*, |
|  |  | boolean | *weighReadCounts*, |
|  |  | String | *strand* |
|  | ) |  | throws IOException |

## Member Function Documentation

|  |  |  |  |  |  |
| --- | --- | --- | --- | --- | --- |
| void broad.pda.seq.segmentation.GenericAlignmentDataModel.clearFullIntervalTreeAsAlignmentsCached | ( | String | *chr* | ) |  |

Implements broad.pda.seq.segmentation.AlignmentDataModel.

|  |  |  |  |  |  |
| --- | --- | --- | --- | --- | --- |
| double broad.pda.seq.segmentation.GenericAlignmentDataModel.countReads | ( | Alignment | *record* | ) |  |

Author
:   : skadri This function will calculate the count value for an alignment read record based on the alue of the weigh ReadCounts flag

Parameters
:   |  |  |
    | --- | --- |
    | record |  |

Returns
:   Count value for alignment

Here is the caller graph for this function:

|  |  |  |  |  |  |
| --- | --- | --- | --- | --- | --- |
| CloseableIterator<Alignment> broad.pda.seq.segmentation.GenericAlignmentDataModel.getAnnotationOverlappingRegion | ( | Annotation | *align* | ) | throws IOException |

Implements broad.pda.seq.segmentation.AlignmentDataModel.

Here is the caller graph for this function:

|  |  |  |  |
| --- | --- | --- | --- |
| int broad.pda.seq.segmentation.GenericAlignmentDataModel.getBasesCovered | ( | Annotation | *region*, |
|  |  | int | *EF* |
|  | ) |  | throws IOException |

Author
:   Jesse Engreitz

Parameters
:   |  |  |
    | --- | --- |
    | region | region of interest |
    | EF | extension factor |

Returns
:   Number of bases covered by at least one read Warning: This is not a very efficient function with lots of reads TODO rewrite using merge

Implements broad.pda.seq.segmentation.AlignmentDataModel.

Here is the call graph for this function:

|  |  |  |  |
| --- | --- | --- | --- |
| double broad.pda.seq.segmentation.GenericAlignmentDataModel.getBasesCoveredPerAlignment | ( | Gene | *gene*, |
|  |  | IntervalTree< Alignment > | *tree*, |
|  |  | int | *EF* |
|  | ) |  |  |

Implements broad.pda.seq.segmentation.AlignmentDataModel.

|  |  |  |  |  |  |
| --- | --- | --- | --- | --- | --- |
| int broad.pda.seq.segmentation.GenericAlignmentDataModel.getChrLength | ( | String | *chr* | ) |  |

Here is the caller graph for this function:

|  |  |  |  |  |
| --- | --- | --- | --- | --- |
| Map<String, Integer> broad.pda.seq.segmentation.GenericAlignmentDataModel.getChrLengths | ( |  | ) |  |

Here is the caller graph for this function:

|  |  |  |  |  |  |
| --- | --- | --- | --- | --- | --- |
| int broad.pda.seq.segmentation.GenericAlignmentDataModel.getChromosomeLength | ( | String | *chr* | ) |  |

Get length of a chromosome

Parameters
:   |  |  |
    | --- | --- |
    | chr |  |

Returns

Implements broad.pda.seq.segmentation.AlignmentDataModel.

|  |  |  |  |  |
| --- | --- | --- | --- | --- |
| Map<String, Integer> broad.pda.seq.segmentation.GenericAlignmentDataModel.getChromosomeLengths | ( |  | ) |  |

Implements broad.pda.seq.segmentation.AlignmentDataModel.

|  |  |  |  |  |  |
| --- | --- | --- | --- | --- | --- |
| CloseableIterator<Alignment> broad.pda.seq.segmentation.GenericAlignmentDataModel.getChromosomeReadIterator | ( | String | *chromosome* | ) | throws IOException |

Parameters
:   |  |  |
    | --- | --- |
    | the | chromosome over which to iterate |

Returns
:   CloseableIterator over all reads mapped to the given Chromosome

Implements broad.pda.seq.segmentation.AlignmentDataModel.

|  |  |  |  |  |  |
| --- | --- | --- | --- | --- | --- |
| CloseableIterator<Alignment> broad.pda.seq.segmentation.GenericAlignmentDataModel.getChrReadIterator | ( | String | *chromosome* | ) | throws IOException |

Here is the call graph for this function:

|  |  |  |  |  |
| --- | --- | --- | --- | --- |
| int broad.pda.seq.segmentation.GenericAlignmentDataModel.getChunkEnd | ( |  | ) |  |

Implements broad.pda.seq.segmentation.AlignmentDataModel.

|  |  |  |  |  |
| --- | --- | --- | --- | --- |
| int broad.pda.seq.segmentation.GenericAlignmentDataModel.getChunkStart | ( |  | ) |  |

Implements broad.pda.seq.segmentation.AlignmentDataModel.

|  |  |  |  |  |  |
| --- | --- | --- | --- | --- | --- |
| double broad.pda.seq.segmentation.GenericAlignmentDataModel.getCounts | ( | String | *chr* | ) | throws IOException |

Counts the number of reads for the specified chromosome

Returns

Exceptions
:   |  |  |
    | --- | --- |
    | IOException |  |

Implements broad.pda.seq.segmentation.AlignmentDataModel.

Here is the call graph for this function:

|  |  |  |  |
| --- | --- | --- | --- |
| double broad.pda.seq.segmentation.GenericAlignmentDataModel.getCountsOfUniqueOverlappers | ( | Annotation | *target*, |
|  |  | Annotation | *exclude*, |
|  |  | IntervalTree< Alignment > | *tree*, |
|  |  | int | *EF* |
|  | ) |  |  |

Implements broad.pda.seq.segmentation.AlignmentDataModel.

|  |  |  |  |
| --- | --- | --- | --- |
| double broad.pda.seq.segmentation.GenericAlignmentDataModel.getCountsOfUniqueOverlappers | ( | Annotation | *target*, |
|  |  | Gene | *exclude*, |
|  |  | IntervalTree< Alignment > | *tree*, |
|  |  | int | *EF* |
|  | ) |  |  |

Implements broad.pda.seq.segmentation.AlignmentDataModel.

|  |  |  |  |
| --- | --- | --- | --- |
| double broad.pda.seq.segmentation.GenericAlignmentDataModel.getCountsOnBase | ( | String | *chr*, |
|  |  | int | *index* |
|  | ) |  | throws IOException |

Implements broad.pda.seq.segmentation.AlignmentDataModel.

Here is the call graph for this function:

|  |  |  |  |
| --- | --- | --- | --- |
| double broad.pda.seq.segmentation.GenericAlignmentDataModel.getCountsOnWindow | ( | String | *chr*, |
|  |  | int | *startIndex*, |
|  |  | int | *endIndex* |
|  | ) |  | throws IOException |

Here is the call graph for this function:

|  |  |  |  |
| --- | --- | --- | --- |
| double broad.pda.seq.segmentation.GenericAlignmentDataModel.getCountsPerAlignment | ( | Annotation | *align*, |
|  |  | int | *EF* |
|  | ) |  | throws IOException |

Implements broad.pda.seq.segmentation.AlignmentDataModel.

Here is the call graph for this function:

Here is the caller graph for this function:

|  |  |  |  |  |  |
| --- | --- | --- | --- | --- | --- |
| double broad.pda.seq.segmentation.GenericAlignmentDataModel.getCountsPerAlignment | ( | Annotation | *align* | ) | throws IOException |

Here is the call graph for this function:

|  |  |  |  |
| --- | --- | --- | --- |
| double broad.pda.seq.segmentation.GenericAlignmentDataModel.getCountsPerAlignment | ( | Annotation | *align*, |
|  |  | IntervalTree< Alignment > | *tree*, |
|  |  | int | *EF* |
|  | ) |  |  |

Implements broad.pda.seq.segmentation.AlignmentDataModel.

Here is the call graph for this function:

|  |  |  |  |
| --- | --- | --- | --- |
| double broad.pda.seq.segmentation.GenericAlignmentDataModel.getCountsPerAlignment | ( | Gene | *gene*, |
|  |  | IntervalTree< Alignment > | *tree*, |
|  |  | int | *EF* |
|  | ) |  |  |

Implements broad.pda.seq.segmentation.AlignmentDataModel.

Here is the call graph for this function:

|  |  |  |  |
| --- | --- | --- | --- |
| double broad.pda.seq.segmentation.GenericAlignmentDataModel.getCountsPerAlignment | ( | Annotation[] | *Annotation*, |
|  |  | IntervalTree< Alignment > | *tree*, |
|  |  | int | *EF* |
|  | ) |  |  |

Implements broad.pda.seq.segmentation.AlignmentDataModel.

|  |  |  |  |
| --- | --- | --- | --- |
| double broad.pda.seq.segmentation.GenericAlignmentDataModel.getCountsPerAlignment | ( | Annotation[] | *Annotation*, |
|  |  | int | *EF* |
|  | ) |  | throws IOException |

Similar to

See also
:   getCountsPerAlignment(Annotation[] Annotation, IntervalTree<Alignment> tree, int EF) but delegates the management of the alignment interval tree to the implementing class rather than the caller

Parameters
:   |  |  |
    | --- | --- |
    | Annotation |  |
    | EF |  |

Returns

Exceptions
:   |  |  |
    | --- | --- |
    | IOException |  |

Implements broad.pda.seq.segmentation.AlignmentDataModel.

|  |  |  |  |
| --- | --- | --- | --- |
| double broad.pda.seq.segmentation.GenericAlignmentDataModel.getCountsPerAlignment | ( | Annotation | *align*, |
|  |  | Map< String, IntervalTree< Annotation >> | *goodExonTree*, |
|  |  | IntervalTree< Alignment > | *tree*, |
|  |  | int | *extensionFactor* |
|  | ) |  |  |

Implements broad.pda.seq.segmentation.AlignmentDataModel.

Here is the call graph for this function:

|  |  |  |  |
| --- | --- | --- | --- |
| double broad.pda.seq.segmentation.GenericAlignmentDataModel.getCountsPerAlignmentForPolyA | ( | Annotation | *align*, |
|  |  | int | *EF* |
|  | ) |  | throws IOException |

|  |  |  |  |
| --- | --- | --- | --- |
| double broad.pda.seq.segmentation.GenericAlignmentDataModel.getCountsPerAlignmentFullyContained | ( | Gene | *gene*, |
|  |  | IntervalTree< Alignment > | *tree*, |
|  |  | int | *EF* |
|  | ) |  |  |

Implements broad.pda.seq.segmentation.AlignmentDataModel.

|  |  |  |  |
| --- | --- | --- | --- |
| double broad.pda.seq.segmentation.GenericAlignmentDataModel.getCountsPerAlignmentUncached | ( | String | *chr*, |
|  |  | int | *startIndex*, |
|  |  | int | *endIndex* |
|  | ) |  | throws IOException |

Here is the caller graph for this function:

|  |  |  |  |
| --- | --- | --- | --- |
| double broad.pda.seq.segmentation.GenericAlignmentDataModel.getCountsPerAlignmentWithSameEndpointForPolyA | ( | Annotation | *align*, |
|  |  | int | *EF*, |
|  |  | boolean | *polyAIsOnRight*, |
|  |  | int | *typicalOriginalAlignmentLength*, |
|  |  | int | *maxDistanceFromOriginalAlignmentEndpoint* |
|  | ) |  | throws IOException |

|  |  |  |  |
| --- | --- | --- | --- |
| double broad.pda.seq.segmentation.GenericAlignmentDataModel.getCountsPerAnnotationtranded | ( | Gene | *gene*, |
|  |  | IntervalTree< Alignment > | *tree*, |
|  |  | int | *EF*, |
|  |  | String | *orientation* |
|  | ) |  |  |

Implements broad.pda.seq.segmentation.AlignmentDataModel.

Here is the call graph for this function:

|  |  |  |  |
| --- | --- | --- | --- |
| int broad.pda.seq.segmentation.GenericAlignmentDataModel.getCountsWithinExons | ( | Annotation | *align*, |
|  |  | IntervalTree< Alignment > | *tree*, |
|  |  | int | *EF* |
|  | ) |  |  |

Implements broad.pda.seq.segmentation.AlignmentDataModel.

Here is the call graph for this function:

|  |  |  |  |
| --- | --- | --- | --- |
| int broad.pda.seq.segmentation.GenericAlignmentDataModel.getCountsWithinExons | ( | Annotation | *align*, |
|  |  | Iterator< Alignment > | *iter*, |
|  |  | int | *EF* |
|  | ) |  |  |

Implements broad.pda.seq.segmentation.AlignmentDataModel.

Here is the call graph for this function:

|  |  |  |  |  |  |
| --- | --- | --- | --- | --- | --- |
| Iterator<Annotation> broad.pda.seq.segmentation.GenericAlignmentDataModel.getExonAnnotationOverlappingRegion | ( | Annotation | *align* | ) | throws IOException |

Implements broad.pda.seq.segmentation.AlignmentDataModel.

Here is the call graph for this function:

|  |  |  |  |  |  |
| --- | --- | --- | --- | --- | --- |
| Iterator<Annotation> broad.pda.seq.segmentation.GenericAlignmentDataModel.getExonAnnotationOverlappingRegion | ( | IntervalTree< Alignment > | *tree* | ) |  |

|  |  |  |  |  |  |
| --- | --- | --- | --- | --- | --- |
| IntervalTree<Annotation> broad.pda.seq.segmentation.GenericAlignmentDataModel.getFullIntervalTreeAsAnnotation | ( | String | *chr* | ) | throws IOException |

Implements broad.pda.seq.segmentation.AlignmentDataModel.

|  |  |  |  |
| --- | --- | --- | --- |
| GeneCounts broad.pda.seq.segmentation.GenericAlignmentDataModel.getGeneCounts | ( | Gene | *gene*, |
|  |  | int | *extensionFactor* |
|  | ) |  | throws IOException |

Implements broad.pda.seq.segmentation.AlignmentDataModel.

Here is the call graph for this function:

|  |  |  |  |
| --- | --- | --- | --- |
| IntervalTree<Alignment> broad.pda.seq.segmentation.GenericAlignmentDataModel.getIntervalTree | ( | String | *chr*, |
|  |  | int | *start*, |
|  |  | int | *end* |
|  | ) |  | throws IOException |

public IntervalTree<Alignment> getIntervalTree(String chr, int start, int end){ System.err.print("Building tree from "+chr+" "+start+"-"+end + " - " ); int counter=0; IntervalTree<Alignment> tree=new IntervalTree<Alignment>(); CloseableIterator<Alignment> iter=getAnnotationOverlappingRegion(new Alignments(chr, start, end)); while(iter.hasNext()){ Alignment record=iter.next(); Annotation t1=new Alignments(record.getChr(), record.getAnnotationtart(), record.getAlignmentEnd()); Annotation t2=new Alignments(chr, start, end); if(t1.overlaps(t2)){ System.err.println("Getting interval overlappers "+start+"-"+end+" "+record.getChr()+":"+record.getAnnotationtart()+"-"+record.getAlignmentEnd());

```
            Node<Alignment> node=tree.find(record.getAnnotationtart(), record.getAlignmentEnd()+1);
            if(record.getMappingQuality()>1){
                if(node!=null){node.incrementCount();}
                else{tree.put(record.getAnnotationtart(), record.getAlignmentEnd()+1, record);}
                counter++;
            }
        }
```

else{System.err.println("WARN: Doesnt overlap queried: "+chr+":"+start+"-"+end+" got "+record.getChr()+":"+record.getAnnotationtart()+"-"+record.getAlignmentEnd());} } iter.close(); System.err.println(tree.size()+" "+counter); return tree; }

Exceptions
:   |  |  |
    | --- | --- |
    | IOException |  |

Implements broad.pda.seq.segmentation.AlignmentDataModel.

Here is the call graph for this function:

Here is the caller graph for this function:

|  |  |  |  |
| --- | --- | --- | --- |
| IntervalTree<Alignment> broad.pda.seq.segmentation.GenericAlignmentDataModel.getIntervalTreeCached | ( | String | *chr*, |
|  |  | int | *start*, |
|  |  | int | *end* |
|  | ) |  | throws IOException |

Implements broad.pda.seq.segmentation.AlignmentDataModel.

Here is the call graph for this function:

Here is the caller graph for this function:

|  |  |  |  |
| --- | --- | --- | --- |
| IntervalTree<Alignment> broad.pda.seq.segmentation.GenericAlignmentDataModel.getIntervalTreeTruncated | ( | String | *chr*, |
|  |  | int | *start*, |
|  |  | int | *end* |
|  | ) |  | throws IOException |

Here is the call graph for this function:

Here is the caller graph for this function:

|  |  |  |  |
| --- | --- | --- | --- |
| IntervalTree<Alignment> broad.pda.seq.segmentation.GenericAlignmentDataModel.getIntervalTreeTruncatedCached | ( | String | *chromosome*, |
|  |  | int | *start*, |
|  |  | int | *end* |
|  | ) |  | throws IOException |

Implements broad.pda.seq.segmentation.AlignmentDataModel.

Here is the call graph for this function:

|  |  |  |  |  |
| --- | --- | --- | --- | --- |
| double broad.pda.seq.segmentation.GenericAlignmentDataModel.getMinimumMappingQuality | ( |  | ) |  |

Implements broad.pda.seq.segmentation.AlignmentDataModel.

|  |  |  |  |  |
| --- | --- | --- | --- | --- |
| String broad.pda.seq.segmentation.GenericAlignmentDataModel.getModelFilePath | ( |  | ) |  |

Implements broad.pda.seq.segmentation.AlignmentDataModel.

|  |  |  |  |  |  |
| --- | --- | --- | --- | --- | --- |
| double broad.pda.seq.segmentation.GenericAlignmentDataModel.getNumberOfSplicedReads | ( | Annotation | *align* | ) | throws IOException |

Implements broad.pda.seq.segmentation.AlignmentDataModel.

Here is the call graph for this function:

|  |  |  |  |  |
| --- | --- | --- | --- | --- |
| double broad.pda.seq.segmentation.GenericAlignmentDataModel.getNumberOfSplicedReads | ( |  | ) |  |

|  |  |  |  |  |  |
| --- | --- | --- | --- | --- | --- |
| Collection<Annotation> broad.pda.seq.segmentation.GenericAlignmentDataModel.getOverlappingRegions | ( | String | *chr* | ) | throws IOException |

Implements broad.pda.seq.segmentation.AlignmentDataModel.

|  |  |  |  |
| --- | --- | --- | --- |
| Gene broad.pda.seq.segmentation.GenericAlignmentDataModel.getPeak | ( | Gene | *gene*, |
|  |  | Gene | *startCodon*, |
|  |  | IntervalTree< Alignment > | *tree*, |
|  |  | int | *EF* |
|  | ) |  |  |

Implements broad.pda.seq.segmentation.AlignmentDataModel.

|  |  |  |  |  |
| --- | --- | --- | --- | --- |
| CloseableIterator<Alignment> broad.pda.seq.segmentation.GenericAlignmentDataModel.getReadIterator | ( |  | ) |  |

Implements broad.pda.seq.segmentation.AlignmentDataModel.

|  |  |  |  |  |  |
| --- | --- | --- | --- | --- | --- |
| CloseableIterator<Alignment> broad.pda.seq.segmentation.GenericAlignmentDataModel.getReadIterator | ( | Annotation | *region* | ) | throws IOException |

Implements broad.pda.seq.segmentation.AlignmentDataModel.

|  |  |  |  |  |
| --- | --- | --- | --- | --- |
| SAMFileHeader broad.pda.seq.segmentation.GenericAlignmentDataModel.getSamHeader | ( |  | ) | throws IOException |

|  |  |  |  |
| --- | --- | --- | --- |
| double broad.pda.seq.segmentation.GenericAlignmentDataModel.getScorePerAlignmentFromCache | ( | Gene | *window*, |
|  |  | IntervalTree< Alignment > | *chunkAlignmentTree*, |
|  |  | int | *extensionFactor* |
|  | ) |  |  |

Implements broad.pda.seq.segmentation.AlignmentDataModel.

|  |  |  |  |  |  |
| --- | --- | --- | --- | --- | --- |
| Collection<Annotation> broad.pda.seq.segmentation.GenericAlignmentDataModel.getSplicedReadExons | ( | String | *chr* | ) | throws IOException |

Implements broad.pda.seq.segmentation.AlignmentDataModel.

Here is the call graph for this function:

|  |  |  |  |  |  |
| --- | --- | --- | --- | --- | --- |
| Map<Annotation, Integer> broad.pda.seq.segmentation.GenericAlignmentDataModel.getSplicedReads | ( | Annotation | *align* | ) | throws IOException |

Implements broad.pda.seq.segmentation.AlignmentDataModel.

Here is the caller graph for this function:

|  |  |  |  |
| --- | --- | --- | --- |
| Map<Annotation, Integer> broad.pda.seq.segmentation.GenericAlignmentDataModel.getSplicedReads | ( | Annotation | *align*, |
|  |  | final int | *minIntronSize*, |
|  |  | final int | *maxIntronSize*, |
|  |  | int | *minNumIntrons*, |
|  |  | Sequence | *chrSeq* |
|  | ) |  | throws IOException |

Implements broad.pda.seq.segmentation.AlignmentDataModel.

Here is the call graph for this function:

|  |  |  |  |
| --- | --- | --- | --- |
| Map<Annotation, Integer> broad.pda.seq.segmentation.GenericAlignmentDataModel.getSplicedReads | ( | Annotation | *align*, |
|  |  | final int | *minIntronSize*, |
|  |  | final int | *maxIntronSize* |
|  | ) |  | throws IOException |

Here is the call graph for this function:

|  |  |  |  |
| --- | --- | --- | --- |
| Map<Annotation, Integer> broad.pda.seq.segmentation.GenericAlignmentDataModel.getSplicedReads | ( | Annotation | *region*, |
|  |  | Predicate< Annotation > | *filter* |
|  | ) |  | throws IOException |

Implements broad.pda.seq.segmentation.AlignmentDataModel.

Here is the call graph for this function:

|  |  |  |  |
| --- | --- | --- | --- |
| Map<Annotation, Integer> broad.pda.seq.segmentation.GenericAlignmentDataModel.getSplicedReads | ( | Annotation | *region*, |
|  |  | Collection< Predicate< Annotation >> | *filters*, |
|  |  | int | *minNumIntrons* |
|  | ) |  | throws IOException |

Implements broad.pda.seq.segmentation.AlignmentDataModel.

Here is the call graph for this function:

|  |  |  |  |
| --- | --- | --- | --- |
| Map<Annotation, Integer> broad.pda.seq.segmentation.GenericAlignmentDataModel.getSplicedReads | ( | Annotation | *region*, |
|  |  | Collection< Predicate< Annotation >> | *filters*, |
|  |  | int | *minNumIntrons*, |
|  |  | Sequence | *chrSeq* |
|  | ) |  | throws IOException |

Implements broad.pda.seq.segmentation.AlignmentDataModel.

Here is the call graph for this function:

|  |  |  |  |
| --- | --- | --- | --- |
| Map<Annotation, Integer> broad.pda.seq.segmentation.GenericAlignmentDataModel.getSplicedReads | ( | Annotation | *region*, |
|  |  | Collection< Predicate< Annotation >> | *filters* |
|  | ) |  | throws IOException |

Implements broad.pda.seq.segmentation.AlignmentDataModel.

Here is the call graph for this function:

|  |  |  |  |
| --- | --- | --- | --- |
| Map<Annotation, Integer> broad.pda.seq.segmentation.GenericAlignmentDataModel.getSplicedReads | ( | Annotation | *region*, |
|  |  | Collection< Predicate< Annotation >> | *filters*, |
|  |  | Sequence | *chrSeq* |
|  | ) |  | throws IOException |

Here is the call graph for this function:

|  |  |  |  |
| --- | --- | --- | --- |
| Map<Annotation, Integer> broad.pda.seq.segmentation.GenericAlignmentDataModel.getSplicedReads | ( | int | *minIntronSize*, |
|  |  | int | *maxIntronSize* |
|  | ) |  | throws IOException |

Implements broad.pda.seq.segmentation.AlignmentDataModel.

Here is the call graph for this function:

|  |  |  |  |  |
| --- | --- | --- | --- | --- |
| double broad.pda.seq.segmentation.GenericAlignmentDataModel.getSpliceWeightFactor | ( |  | ) |  |

Implements broad.pda.seq.segmentation.AlignmentDataModel.

|  |  |  |  |  |
| --- | --- | --- | --- | --- |
| double [] broad.pda.seq.segmentation.GenericAlignmentDataModel.getTotalNumberOfMappedReads | ( |  | ) |  |

|  |  |  |  |  |
| --- | --- | --- | --- | --- |
| double [] broad.pda.seq.segmentation.GenericAlignmentDataModel.getTotalNumberOfMappedStrandedReads | ( |  | ) |  |

|  |  |  |  |  |
| --- | --- | --- | --- | --- |
| double broad.pda.seq.segmentation.GenericAlignmentDataModel.getTotalNumberOfReads | ( |  | ) |  |

|  |  |  |  |  |
| --- | --- | --- | --- | --- |
| double broad.pda.seq.segmentation.GenericAlignmentDataModel.getTotalNumberOfStrandedReads | ( |  | ) |  |

Implements broad.pda.seq.segmentation.AlignmentDataModel.

|  |  |  |  |  |
| --- | --- | --- | --- | --- |
| double broad.pda.seq.segmentation.GenericAlignmentDataModel.getWeight | ( |  | ) | throws IOException |

|  |  |  |  |  |  |
| --- | --- | --- | --- | --- | --- |
| boolean broad.pda.seq.segmentation.GenericAlignmentDataModel.hasNextExon | ( | String | *chr* | ) | throws IOException |

Implements broad.pda.seq.segmentation.AlignmentDataModel.

Here is the call graph for this function:

|  |  |  |  |  |
| --- | --- | --- | --- | --- |
| boolean broad.pda.seq.segmentation.GenericAlignmentDataModel.isNegativeStranded | ( |  | ) |  |

Implements broad.pda.seq.segmentation.AlignmentDataModel.

Here is the call graph for this function:

|  |  |  |  |  |
| --- | --- | --- | --- | --- |
| boolean broad.pda.seq.segmentation.GenericAlignmentDataModel.isPositiveStranded | ( |  | ) |  |

Implements broad.pda.seq.segmentation.AlignmentDataModel.

Here is the call graph for this function:

|  |  |  |  |  |
| --- | --- | --- | --- | --- |
| boolean broad.pda.seq.segmentation.GenericAlignmentDataModel.isStranded | ( |  | ) |  |

Implements broad.pda.seq.segmentation.AlignmentDataModel.

Here is the call graph for this function:

Here is the caller graph for this function:

|  |  |  |  |  |
| --- | --- | --- | --- | --- |
| boolean broad.pda.seq.segmentation.GenericAlignmentDataModel.loadedPairsAsFragments | ( |  | ) |  |

|  |  |  |  |
| --- | --- | --- | --- |
| boolean broad.pda.seq.segmentation.GenericAlignmentDataModel.passes | ( | Gene | *gene*, |
|  |  | IntervalTree< Alignment > | *tree*, |
|  |  | int | *EF*, |
|  |  | double | *cutoff* |
|  | ) |  |  |

Implements broad.pda.seq.segmentation.AlignmentDataModel.

Here is the call graph for this function:

Here is the caller graph for this function:

|  |  |  |  |
| --- | --- | --- | --- |
| long broad.pda.seq.segmentation.GenericAlignmentDataModel.passesCutoff | ( | Annotation | *align*, |
|  |  | IntervalTree< Alignment > | *tree*, |
|  |  | int | *EF*, |
|  |  | double | *threshold* |
|  | ) |  |  |

Implements broad.pda.seq.segmentation.AlignmentDataModel.

Here is the call graph for this function:

|  |  |  |  |  |  |
| --- | --- | --- | --- | --- | --- |
| void broad.pda.seq.segmentation.GenericAlignmentDataModel.resetTreeCache | ( | String | *chr* | ) |  |

Implements broad.pda.seq.segmentation.AlignmentDataModel.

|  |  |  |  |  |
| --- | --- | --- | --- | --- |
| void broad.pda.seq.segmentation.GenericAlignmentDataModel.resetTreeCache | ( |  | ) |  |

Implements broad.pda.seq.segmentation.AlignmentDataModel.

Here is the caller graph for this function:

|  |  |  |  |  |
| --- | --- | --- | --- | --- |
| void broad.pda.seq.segmentation.GenericAlignmentDataModel.restartIterator | ( |  | ) |  |

Implements broad.pda.seq.segmentation.AlignmentDataModel.

|  |  |  |  |  |  |
| --- | --- | --- | --- | --- | --- |
| void broad.pda.seq.segmentation.GenericAlignmentDataModel.setChunkSize | ( | int | *chunkSize* | ) |  |

Implements broad.pda.seq.segmentation.AlignmentDataModel.

|  |  |  |  |  |  |
| --- | --- | --- | --- | --- | --- |
| void broad.pda.seq.segmentation.GenericAlignmentDataModel.setExtensionFactor | ( | int | *extensionFactor* | ) |  |

Implements broad.pda.seq.segmentation.AlignmentDataModel.

|  |  |  |  |  |
| --- | --- | --- | --- | --- |
| void broad.pda.seq.segmentation.GenericAlignmentDataModel.setFirstRead | ( |  | ) |  |

Implements broad.pda.seq.segmentation.AlignmentDataModel.

|  |  |  |  |  |  |
| --- | --- | --- | --- | --- | --- |
| void broad.pda.seq.segmentation.GenericAlignmentDataModel.setMinimumMappingQuality | ( | double | *minimumMappingQual* | ) |  |

Implements broad.pda.seq.segmentation.AlignmentDataModel.

|  |  |  |  |  |
| --- | --- | --- | --- | --- |
| void broad.pda.seq.segmentation.GenericAlignmentDataModel.setNegativeStranded | ( |  | ) |  |

Implements broad.pda.seq.segmentation.AlignmentDataModel.

|  |  |  |  |  |  |
| --- | --- | --- | --- | --- | --- |
| void broad.pda.seq.segmentation.GenericAlignmentDataModel.setNormalizationStrategy | ( | ReadCountNormalizer | *normalizer* | ) |  |

Implements broad.pda.seq.segmentation.AlignmentDataModel.

|  |  |  |  |  |
| --- | --- | --- | --- | --- |
| void broad.pda.seq.segmentation.GenericAlignmentDataModel.setPositiveStranded | ( |  | ) |  |

Implements broad.pda.seq.segmentation.AlignmentDataModel.

|  |  |  |  |  |  |
| --- | --- | --- | --- | --- | --- |
| void broad.pda.seq.segmentation.GenericAlignmentDataModel.setRemoveDuplicatesFlag | ( | boolean | *flag* | ) |  |

Here is the caller graph for this function:

|  |  |  |  |  |
| --- | --- | --- | --- | --- |
| void broad.pda.seq.segmentation.GenericAlignmentDataModel.setSecondRead | ( |  | ) |  |

Implements broad.pda.seq.segmentation.AlignmentDataModel.

|  |  |  |  |  |  |
| --- | --- | --- | --- | --- | --- |
| void broad.pda.seq.segmentation.GenericAlignmentDataModel.setWeighReadCountsFlag | ( | boolean | *flag* | ) |  |

Author
:   : skadri This function will set the weighReadCounts flag which decides how the reads are counted for each alignment

Parameters
:   |  |  |
    | --- | --- |
    | flag | true if the read counts should be weighed by the value of the NH flag |

Here is the caller graph for this function:

|  |  |  |  |  |
| --- | --- | --- | --- | --- |
| void broad.pda.seq.segmentation.GenericAlignmentDataModel.unsetStranded | ( |  | ) |  |

Implements broad.pda.seq.segmentation.AlignmentDataModel.

Here is the call graph for this function:

|  |  |  |  |
| --- | --- | --- | --- |
| Gene broad.pda.seq.segmentation.GenericAlignmentDataModel.updateGeneByFirstCounts | ( | Gene | *gene*, |
|  |  | IntervalTree< Alignment > | *tree*, |
|  |  | int | *EF* |
|  | ) |  |  |

Implements broad.pda.seq.segmentation.AlignmentDataModel.

---

The documentation for this class was generated from the following file:

- java/broad/pda/seq/segmentation/GenericAlignmentDataModel.java


---

Generated on Fri Aug 15 2014 15:11:04 for ESAT by  

 1.8.7
